# Supplementary material for: Protein FID: improved evaluation of protein structure generative models
Source: Bioinformatics. 2026 Apr 2;42(4):btag156. doi: 10.1093/bioinformatics/btag156 (PMC13092321; doi:10.1093/bioinformatics/btag156)
Supplement: btag156_Supplementary_Data [file btag156_supplementary_data.pdf]

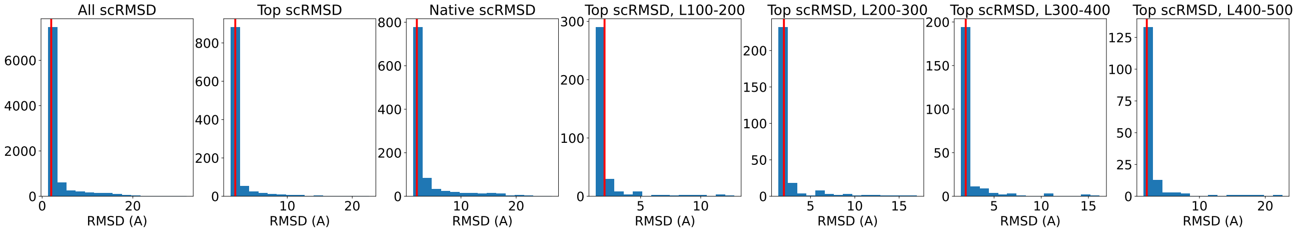

Fig. 11. PDB scRMSDs. We plot the distribution of scRMSDs for the PDB set from Fig. 1. The scRMSD is the RMSD between the structure, and the ESMFold structure prediction using either the native sequence, or a sequence from PMPNN. We sample 8 sequences from PMPNN for each structure. We show the distribution of all scRMSDs (all 8 PMPNN sequences), the top scRMSDs (lowest out of 8), the native scRMSDs, and the top scRMSDs broken down by the same length ranges as in Fig. 1. The red line shows the 2Åcutoff used to consider a structure designable.

Appendix A Additional Figures

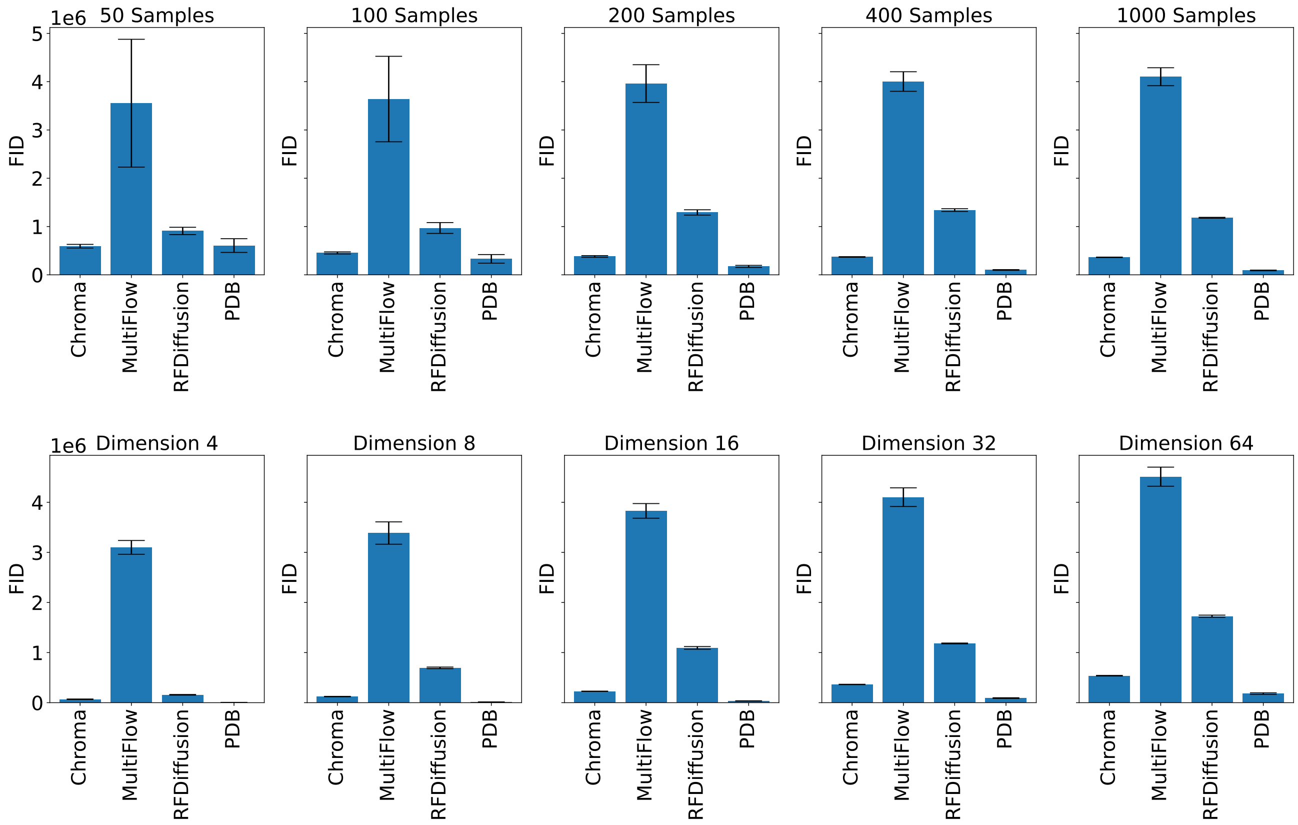

Fig. 12. Robustness Analysis *Top*: FIDs of generative models for different numbers of samples. *Bottom*: FIDs of generative models for different dimensions of embeddings. In all cases, we see that the order of the models is preserved.

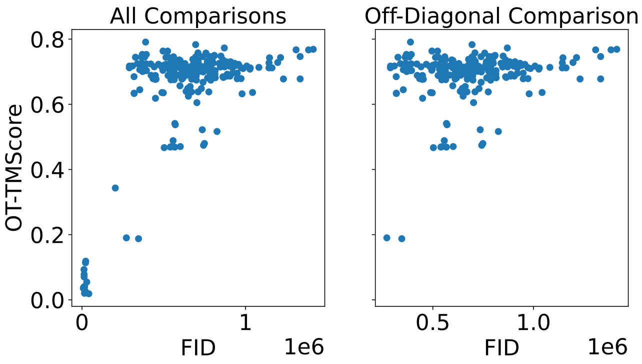

Fig. 13. Scatter plot of cluster comparisons. We plot the OT-TMScore against the FID for the cluster comparisons in Fig. 3. The left panel shows all comparisons, and the right panel only shows the the off-diagonal comparisons between samples from different clusters. Respective Peasron correlations are $r = 0.61 (p = 1.94 \times 10^{-22}\%)$ and $r = 0.2 (p = 0.28\%)$.

Appendix B Comparison of Embedding Methods

Here we explore other ways of computing the embeddings. We consider using ESM3 [Hayes et al., 2024] as an alternative to GearNet. ESM3 is a protein language model that also encodes protein structure via a structure tokenizer. We computing ESM3 embeddings, we mask out the sequence so that the embeddings only capture the structure. We average the residue embeddings to obtain a protein representation. We also explore using the residue representations directly. In this case, the FID is computed between all the residue embeddings in the reference set and the embeddings in the generated set.

We first illustrate that for both ESM3 and GearNet, the embedded distributions have extremely rank-deficient covariance matrices, motivating the need to project the embeddings to a lower dimension. We then present results for all the same experiments as in the main text, but with GearNet embeddings replaced by ESM3 embeddings. We will show that the FIDs computed using ESM3 embeddings do not behave as cleanly as those computed using GearNet embeddings. Next, we consider using the residue embeddings from ESM3 instead of using the average embedding and see that the resulting FID focuses more on local rather than global structure.

Degeneracy of Embeddings

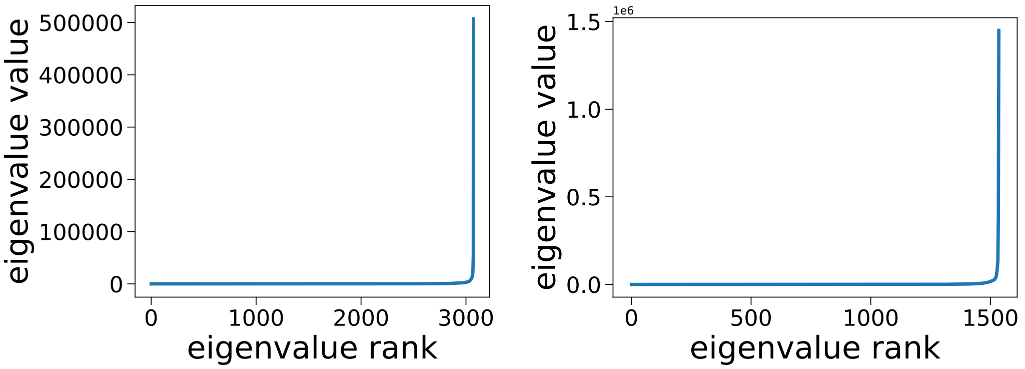

Fig. 14. Degeneracy of Embeddings Sorted eigenvalues of covariance matrix of embedded distribution. *Left*: Embeddings from GearNet. *Right*: Embeddings from ESM3.

For both GearNet and ESM3 we compute the embeddings of our reference set. We then compute the eigenvalues of the covariance matrix of the resulting embeddings, which we plot in sorted order in Fig. 14. In both cases we can see from the small number of dominating eigenvalues that the embedded distributions are spread over a very low-dimensional subspace. We believe this to be the cause of numerical instabilities we observed when trying to compute FIDs using the full dimensional embeddings. This motivated our approach of first projecting the embeddings to a lower dimensional subspace.

ESM3 Embeddings

In addition to GearNet, we also explore using ESM3 [Zhang et al., 2022] to compute embeddings. As can be seen in Fig. 14, we also find the need to project the embeddings down to a lower dimension, using the same approach as for GearNet. Using these embeddings to compute FIDs, we report the same experiments as for GearNet, including FIDs of perturbations (Fig. 15), FIDs between FoldSeek Clusters (Fig. 16, and diversity races (Fig .17). Overall, we find that the ESM3-based FID is very sensitive to

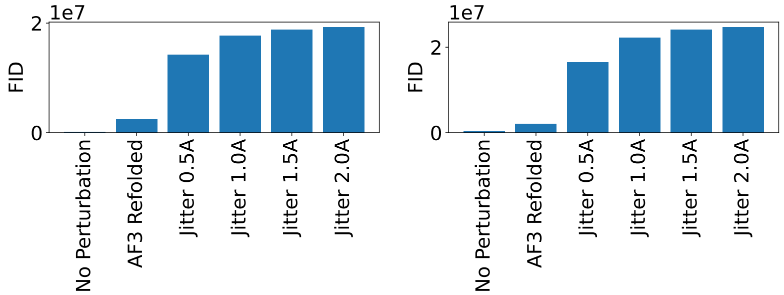

Fig. 15. FID Ladders. FIDs of perturbed PDB samples for averaged ESM3 (left) and split ESM3 embeddings (right).

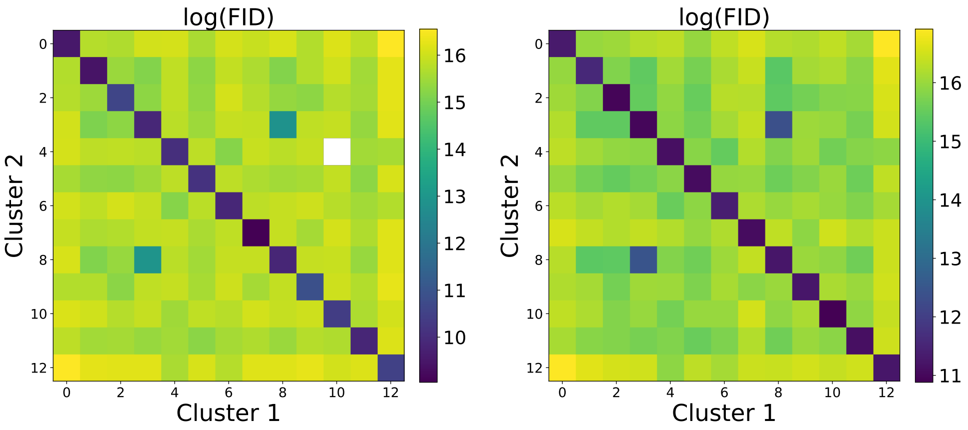

Fig. 16. FID Between FoldSeek Clusters *Left:* Pairwise FIDs between FoldSeek clusters computed with averaged ESM3 embeddings. Correlation with OT-TMScores is 0.66. *Right:* Pairwise FIDs between FoldSeek clusters computed with split ESM3 embeddings. Correlation with OT-TMScores is 0.7.

small perturbations of the structure. For example, adding 0.5Å of random noise to PDB structures gives a much higher FID than AF3-refolded structures. We also observe this when evaluating generative models, which achieve a much higher FID than the PDB test set. Generative models are known to have issues reproducing correct bond lengths and we suspect that the ESM3 embeddings penalize this heavily. This is further illustrated in Fig. 18, where we see that at only 0.5Åof random noise, the PDB structures are already completely out of distribution.

Split ESM3 Embeddings

ESM3 computes embeddings for each residue in a protein, which we average to compute a whole-protein representation. Instead, we also explore keeping the residue embeddings separate and computing FIDs using the pooled embeddings. This could focus the FID on differences in local structure rather than global structure. Hence, if two sets of structures can be decomposed into similar sets of *local* structures, they would still achieve a low FID even if the *global* structures are different. Because the total number of residues in a set of protein structures can grow very large, we subsample residue embeddings when computing the FIDs. We also still project the embeddings to a lower dimension. Just as for averaged ESM3, we reproduce the same experiments as in the main paper but using these split embeddings. Interestingly, we see that the FID of AF3-refolded samples in Fig. 15 is closer to the PDB samples when compared to using averaged ESM3 embeddings. The ratio of AF3-refolded to PDB FIDs is around 19 when using averaged embeddings, and only around 6 with split embeddings. We hypothesize that, without access to MSAs, AlphaFold3 is still able to reconstruct local structures faithfully, although it may fail to produce accurate global structures. This suggests that the split ESM3 embeddings indeed focus more on local differences than global differences. This may be attractive in some cases, although for the purposes of our experiments we find that overall the averaged embeddings still perform better when looking at the inter-cluster FIDs (Fig. 16) and diversity races (Fig. 17).

Appendix C Embedding Dimension for GearNet

We found it useful to project the GearNet embeddings to a lower dimension using PCA. Here we perform a sweep of the embedding dimension, reporting results when using different numbers of embeddings. Fig. 19 shows the CATH diversity races when run using

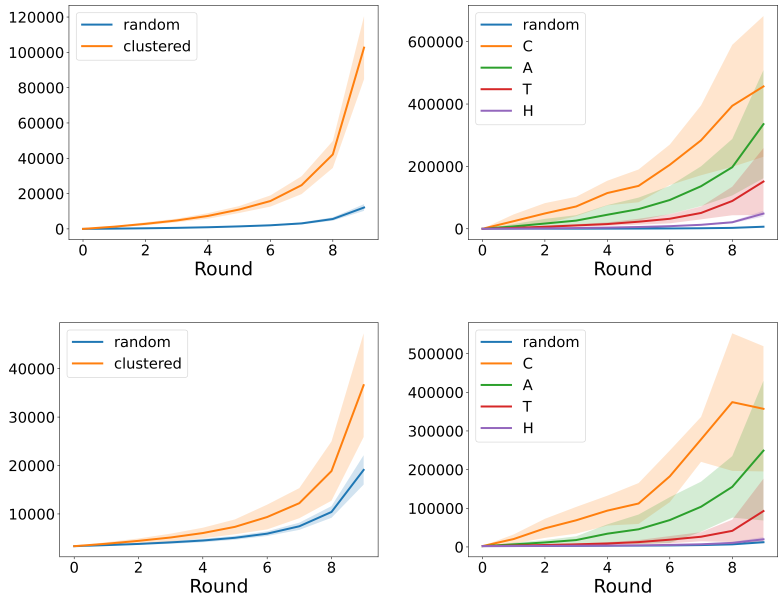

Fig. 17. Diversity races. *Top row*: Diversity races conducted using averaged ESM3 embeddings. *Bottom row*: Diversity races conducted using split ESM3 embeddings. *Left*: Race conducted with FoldSeek clusters. *Right*: Race conducted with CATH clusters, with one racer for each level of the hierarchy.

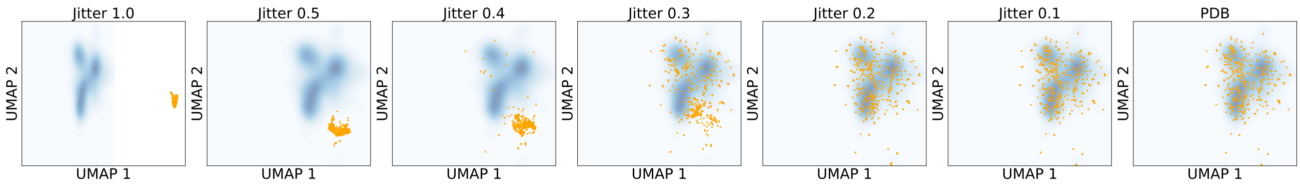

Fig. 18. Effect of noisy structures on ESM3 embeddings. We apply small amounts of random noise to the coordinates of the PDB test set (0.1-1.0Å) and plot the 2-dimensional UMAP projections of the resulting averaged ESM3 embeddings. We see that at 0.5Åof noise, the structures are already completely out of distribution.

different embedding dimensions. We see that if we project onto too few dimensions, we start to lose the separation and ordering of the racers. We thus opted to use 32 dimensions since this was the lowest dimension that showed good results in the CATH race, and it also did not lead to any numerical issues when computing FIDs.

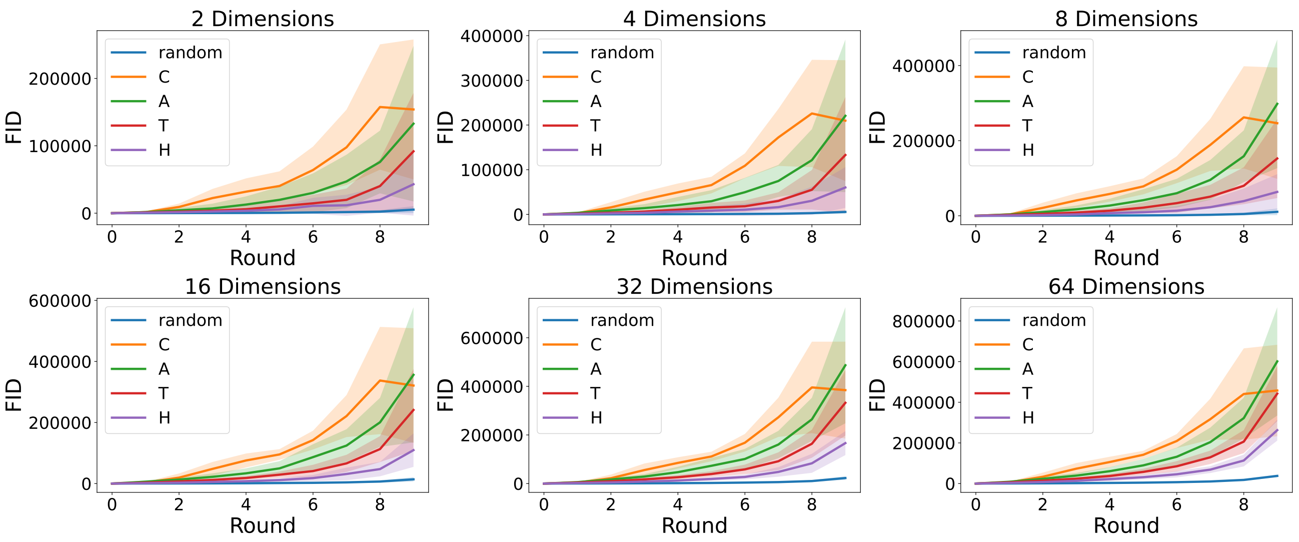

Fig. 19. Diversity Races with Varying Dimension CATH diversity races for different embedding dimensions.
